# Can Integrated Watershed Management Contribute to Improvement of Public Health? A Cross-Sectional Study from Hilly Tribal Villages in India

**DOI:** 10.3390/ijerph120302653

**Published:** 2015-02-27

**Authors:** Sandeep S. Nerkar, Ashish Pathak, Cecilia Stålsby Lundborg, Ashok J. Tamhankar

**Affiliations:** 1 Department of Public Health Sciences, Global Health (IHCAR), Karolinska Institutet, Stockholm, SE 171 77, Sweden; E-Mails: ashish.pathak@ki.se (A.P.); Cecilia.Stalsby.Lundborg@ki.se (C.S.L.); ejetee@gmail.com (A.J.T.); 2 Department of Environmental Medicine, Indian Initiative for Management of Antibiotic Resistance, R. D. Gardi Medical College, Ujjain 456010, India; 3 Department of Pediatrics, R. D. Gardi Medical College, Ujjain 456010, India; 4 Department of Women and Children’s Health, International Maternal and Child Health Unit, Uppsala University, Uppsala, SE 751 85, Sweden

**Keywords:** integrated watershed management, public health, tribal villages

## Abstract

Tribal people living in hilly areas suffer from water scarcity in many parts of the world, including India. Water scarcity adversely impacts all aspects of life, including public health. Implementation of an Integrated Watershed Management Programme (IWMP) can help solve the problems arising out of water scarcity in such areas. However, the knowledge about and views of the water scarcity sufferers on the public health implications of IWMP have not been well documented. This cross-sectional study was performed in six purposively selected tribal villages located in Maharashtra, India. In three of the villages IWMP had been implemented (IWMV), but not in the other three (NWMV). The head of each household in all villages was interviewed using a questionnaire covering various public health aspects relevant to the villages. A total of 286/313 (92%) households participated in the study. Compared to NWMV, respondents in IWMV experienced significantly lesser prolonged water scarcity (OR = 0.39), had greater number of toilets (OR = 6.95), cultivated more variety of crops (OR = 2.61), had lower migration (OR = 0.59), higher number of girls continuing education (OR = 3.04) and better utilized modern healthcare facilities in the antenatal, natal and postnatal period (OR = 3.75, 2.57, 4.88 respectively). Thus, tribal people in IWMP-implemented villages reported advantages in many aspects of public health.

## 1. Introduction

Globally around 1.2 billion people are affected by water scarcity and it is estimated that by 2025, this number will increase by 600 million more people [1]. Lack of water adversely impacts hygiene and sanitation practices [2]. Poor hygiene and sanitation lead to repeated epidemics of water-borne diseases [3]. Also, in arid parts of the world, women have to spend significant productive time and efforts to collect water for daily needs [4]. Scarcity of water for agriculture, reduces crop diversity and agricultural output, thus reduces income which in turn leads to secondary health impacts, such as malnutrition and reduced cognitive function in children [2]. This was recently recognized of importance by Bill and Melinda Gates Foundation which announced, “All children thriving (healthy physical growth and cognitive development in children)” and “putting women and girls at the center of development”, as new initiatives in Grand Challenges in Global Health, 2014 [5]. Improving global access to clean water and safe sanitation is probably the most effective means to improve public health and save lives and is also important for sustainable development [6,7]. 

In many parts of the world, as in rural India, declining groundwater is a major concern for development, as the livelihood of a majority of the rural population depends on agriculture and availability of sustainable water resources [8]. More productive use of rainwater in arid regions of the world, including India, is necessary to help mitigate the impact of water scarcity [9]. An integrated watershed management programme (IWMP) plays a significant role in building resilience in arid rural areas by improved water availability, increased food production, improved livelihood, protection of the agro-ecosystem, addressing gender issues and generation of social capital and economic benefits for the rural population [10].

Considering the benefits of IWMP, the Government of India formulated guidelines for the implementation of an IWMP, which focus not only on soil and water conservation, but also suggest to converge all relevant developmental schemes such as plantation of trees, improving agriculture, formation of self-help groups, improving hygiene and sanitation, and management of drinking water [11]. This paper is about the IWMP implemented at village or micro-watershed level in India which ensures participation of people living there. In India such programmes are promoted by government and non-government organisations (NGOs) with the aim of achieving sustainable development [12]. These various aspect of an IWMP are directly and indirectly linked to health and therefore it is interesting to explore the impact of an IWMP on public health, particularly because comprehensive and scientific information on the linkages between public health and watershed management are poorly integrated [13,14]. This is more so with respect to vulnerable regions like tribal areas of India that are hilly inaccessible lands in the forests with recurrent water scarcity. The concept of the watershed governance prism proposed by Parkes *et al.* suggests an integrative framework to link social and environmental determinants of health in the context of watershed [15]. Further, a qualitative study done by our research group, suggests that IWMP was perceived by the tribal people as a measure that empowered them and improved their health and socio-economic condition [16]. We therefore conducted a quantitative cross-sectional study that aimed to explore the potential advantages of IWMP, on various aspects of public health as perceived by the people concerned and to compare the reported situation with regards to various aspects of public health between two settings, with and without implementation of IWMP.

## 2. Experimental Section 

### 2.1. Study Area, Setting and Design

The study was carried out in six villages located in hilly terrain in a rural Jawhar block of the Thane district in the state of Maharashtra in Western India. The Jawhar block is located approximately 150 km to the north of Mumbai city and has 90% tribal population [17]. In the selected study villages, all people were tribal in origin. The literacy rate of the rural Jawhar block is 45%, which is only about half of the overall literacy rate of the Thane district (81%) [17]. The Jawhar block is located in the tropical climatic zone of India and has three distinctive weather seasons in a year *i.e.*, a rainy season (June to September), a winter season (October to January) and a summer season (February to May). The area receives approximately 2500 mm of rainfall per annum, mainly in the rainy season [18]. Despite receiving adequate rainfall, the tribal people in this area face water scarcity in the non-rainy seasons of the year. The livelihood of the tribal people residing in this area is dependent on rain-fed agriculture and forest produce. For healthcare services, people are dependent on government (Maharashtra)-run primary health centers (PHC), but some people also seek help from traditional healers called *Bhagat* by the community.

This cross-sectional study was carried out in 2011. Six villages were chosen purposively, on the basis of implementation of the integrated watershed management programme (IWMP). In three villages, the IWMP had been implemented (integrated watershed management villages—IWMV) while in the remaining three villages, it had not been implemented (non-watershed management villages—NWMV). All the households (n = 313) in the six selected villages were included in the study. The populations of the IWMV and NWMV groups were 663 and 639 inhabitants, respectively.

### 2.2. Operational Definitions 

For the study, water scarcity was defined as a perceived lack of water for daily needs. Water scarcity was considered as prolonged if it exceeded more than four consecutive months in a year. A physically accessible water source was defined according to the World Health Organization (WHO) definition, as a source that was located within 1000 m of the household and had a water collection time that did not exceed 30 min [19]. Migration was defined as the process of “moving of people” in response to availability of employment as laborer or in response to climate conditions [20]. Diarrhoea was defined as the passage of three or more loose or liquid stools per day [21]. According to WHO, public health refers to all organized measures (whether public or private) to prevent disease, promote health, and prolong life among the population as a whole [22].

### 2.3. Integrated Watershed Management

In the three IWMV, the implementation of IWMP had been undertaken in three phases. The first “preparatory phase”, undertook urgent works to identify and repair water structures, create awareness regarding soil and water conservation and encourage community participation. In the second “work phase”, soil and water conservation was done to reduce the surface water runoff along with plantation of tree crops and development of low-cost water harvesting structures such as check dams and groundwater well recharge. Interventions to improve crop cultivation, grow fruit orchards and pasture development were also undertaken. Building of toilets was promoted and subsidized by integrating “Total Sanitation Campaign” of Government of India [23] with the IWMP. In the third “consolidation phase”, activities that were considered important by the local community like water conservation, management of drinking water resources, improvement of crops and development of fruit orchards and building of toilets were scaled up. In IWMV, it took about three to five years to implement the abovementioned phases in each village. The implementation of the IWMP in the study villages was done by an NGO. According to Kerr, there was a greater success to the integrated watershed management projects implemented at micro-watershed or village level by NGOs [12]. In such projects, there was more focus on social organizations. For this study, in the selected IWMV, people’s participation in the programme was an important feature.

### 2.4. Data Collection Tool

A structured questionnaire to be used in face-to-face interviews was developed based on literature search and consultations done with experts in the field of integrated watershed management to understand the perceived influence of integrated watershed management on public health. The questionnaire included questions on demographic data, water availability, cropping pattern, staple food, hygiene and sanitation, diseases, utilization of healthcare services and knowledge about watershed management. A 10-item part of the questionnaire, with “yes” or “no” answers, was also included to capture perceptions on impact of IWMP on public health, which was based on the results from the qualitative study done earlier in the same area [16]. The questionnaire was pilot tested in 20 households for feasibility and face validity before the actual study.

### 2.5. Data Collection Method 

The data collection was done with the help of study assistants, who belonged to the tribal community, but not the study villages. They were students in the discipline of rural development in a nearby town. The study assistants were therefore well aware of the local language, traditions and practices that the local community followed. They were further trained during the pilot study to conduct interviews. The head of each household, male or female was interviewed. If the interviewee was unaware of IWMP activities, a short explanation about IWMP was given with an example of a village where IWMP had been implemented. In each village, all the interviews were conducted on the same day to avoid discussion among the villagers about the interview. Along with the interview, drinking water samples from each household were tested for potability using a commercially available hydrogen sulfide test kit (Rapid H_2_S kit-Microexpress, Tulip, Goa, India). The manufacturer’s instructions were followed; 20 mL of water was collected from the storage container of each household and tested for faecal coliform contamination [24]. For diarrhoea, repeated cross-sectional data was collected three times in a year, at an interval of four months by the study assistants. Each household was asked about the occurrence of diarrhoea among the household members in the past 15 days.

### 2.6. Data Management and Analysis 

The data was double entered and cross-checked in Microsoft Excel and analyzed using Stata 12.0 (Stata Corp., College Station, TX, USA). Descriptive statistics were used to present the data. Pearson’s chi-square test was used to test statistical significance as applicable. The relationship between each variable and the outcome was explored using odds ratio (OR) estimated with 95% confidence interval (CI). A value of *p* < 0.05 was considered statistically significant.

### 2.7. Ethical Approval

All subjects gave their informed consent for inclusion before they participated in the study. The protocol of the study was approved by the Ethics Committee of the R. D. Gardi Medical College, Ujjain, India (No. 175/2011). 

## 3. Results and Discussion

### 3.1. Details of the Interviewee 

Interviews could be completed in 286 (92%) households out of the total of 313 households in the six villages (142 in IWMV and 144 in NWMV). Among the 27 households, where interviews could not be conducted, a majority (n = 24) of the houses were found closed, while three households did not agree to participate in the study. There was no statistically significant difference in the proportion of participating households (IWMV—93% *vs.* NWMV—91%, *p* = 0.354) or in the characteristics of household heads that participated in the study from IWMV (n = 142) or NWMV (n = 144). The mean age of the household heads was 35 years (SE ± 0.79). The family size of most of the households (n = 164; 57%) was between three to five members. Table 1 gives the details of the characteristics of the household heads (interviewees).

**Table 1:** Gender, education and occupation of the interviewees in six tribal villages, three with and three without implementation of integrated watershed management programme in Thane district, Maharashtra, India.

Variable	IWMV (N = 142) Number (%)	NWMV (N = 144) Number (%)	*p*-Value *
*Gender*			
Male	65 (46)	75 (52)	0.286
Female	77 (54)	69 (48)	
*Education*			
No formal education	85 (60)	94 (65)	0.189
Primary	8 (6)	14 (10)	
Secondary	16 (11)	15 (10)	
Higher Secondary & above	33 (23)	21 (15)	
*Occupation ^#^*			
Farming	96 (68)	103 (72)	0.795
Farming and Labourer	28 (20)	27 (19)	
Labourer	12 (8)	9 (6)	
Farming and other	6 (4)	5 (3)	

N—Total number of interviewees; IWMV—Integrated watershed management villages; NWMV—Non-watershed management villages; * Chi-square test; **^#^** Occupation of the people in households.

### 3.2. Impact of IWMP

Table 2 shows the reported impact of IWMP on water availability, hygiene and sanitation, agricultural practices, migration, and utilization of healthcare services. The details are presented hereafter.

**Table 2:** Differences in variables reported by the interviewees in six tribal villages, three with and three without implementation of integrated watershed management programme in Thane district, Maharashtra, India.

Variable		IWMV (N = 142) Number (%)	NWMV (N = 144) Number (%)	Odds Ratio	95% Confidence Interval	*p*-Value *
Water availability					
Prolonged scarcity of water **^#^**	Yes	20 (14)	126 (88)	0.02	0.01–0.04	<0.001
	No	122 (86)	18 (12)	Ref		
Average distance of water source <1000 m during scarcity period	Yes	123 (87)	103 (71)	2.57	1.42–4.66	0.002
	No	19 (13)	41 (29)	Ref		
Hygiene and sanitation								
Bathing place	Home	85 (60)	59 (41)	2.14	1.34–3.43	0.001
	River	57 (40)	85 (59)	Ref		
Washing of clothes	Home	53 (38)	23 (16)	3.13	1.81–5.42	<0.001
	River	89 (62)	121 (84)	Ref		
Practice of defaecation	Toilet	79 (56)	22 (15)	6.95	4.07–11.85	<0.001
	Open air	63 (44)	122 (85)	Ref		
Need of disinfection of water source	Throughout the year	120 (85)	95 (66)	2.81	1.60–4.92	<0.001
	Only in rainy season	22 (15)	49 (34)	Ref		
Faecal contamination of drinking water at household level (H_2_S test) **^a,π^**	No contamination	82 (58)	17 (12)	10.20	5.83–17.85	<0.001
	Contamination	60 (42)	127 (88)	Ref		
Agriculture and food								
Growing of fruits and vegetable crops **^b^**	Yes	77 (59)	48 (35)	2.61	1.60–4.27	<0.001
	No	54 (41)	88 (65)	Ref		
Rice as staple food	Yes	108 (76)	80 (56)	2.02	1.21–3.36	0.007
	No	34 (24)	64 (44)	Ref		
Vegetable consumption (after purchase)	Yes	110 (77)	96 (67)	1.71	1.01–2.89	0.041
	No	32 (23)	48 (33)	Ref		
Fruits consumption (after purchase)	Yes	63 (44)	42 (29)	1.94	1.19–3.15	0.007
	No	79 (56)	102 (71)	Ref		
Migration	Yes	86 (61)	104 (72)	0.59	0.36–0.97	0.036
	No	56 (39)	40 (28)	Ref		
**Discontinuation of girl-child education**		**(N = 90)** **Number (%)**	**(N = 87)** **Number (%)**			
	No discontinuation	80 (89)	63 (72)	3.04	1.28–7.64	0.005
	Discontinuation	10 (11)	24 (28)	Ref		

N—Total number of interviewees; IWMV—Integrated watershed management villages; NWMV—Non-watershed management villages; Ref—reference category; * Chi-square test; ^#^ Prolonged scarcity period—scarcity of water for more than 4 months in a year; H_2_S test—hydrogen sulfide test to detect the faecal coliform contamination in water; **^a^** Total number of households for NWMV = 140; **^b^** Total number of households for IWMV = 131 and NWMV = 136; **^π^** Tested for households.

#### 3.2.1. Water Availability

All the respondents from the six villages reported water scarcity in their village during some part or parts of the year. The duration of water scarcity differed between IWMV and NWMV. A significantly lower number of households from IWMV (n = 20) reported having prolonged water scarcity compared to households from NWMV (n = 126), (OR = 0.02, 95% CI 0.01–0.04; *p* < 0.001). Further, a significantly higher number of households from IWMV reported a physically accessible water source (less than 1000 m from a house) to be available during scarcity period compared to NWMV (n = 123 *vs.* n = 103, OR = 2.57, 95% CI 1.42–4.66; *p* = 0.002).

#### 3.2.2. Hygiene, Sanitation and Diarrhoea

##### Bathing and Washing of Clothes

A significantly higher number of households from IWMV (85/142; 60%) reported taking bath at home compared to NWMV (59/144; 41%) (OR = 2.14, 95% CI 1.34–3.43; *p* = 0.001). The remaining households in both settings used the river for bathing. The river was also the most common place for washing of clothes in both settings but a significantly higher number of households in IWMV (53/142; 38%) had other options like a well, check dams or ponds as compared to NWMV (23/144; 16%) (OR = 3.13, 95% CI 1.81–5.42; *p* < 0.001).

##### Defaecation Practice and Toilet Use

A significantly higher number of households reported use of toilets for defaecation in IWMV (79/142; 56%), compared to NWMV (22/144; 15%) (OR = 6.95, 95% CI 4.07–11.85; *p* < 0.001). Open air defaecation was a common practice in most (122/144; 85%) of the households in NWMV. There were a significantly higher numbers of households with toilets in IWMV (83/142; 58%) compared to NWMV (27/144; 19%) (OR = 6.09, 95% CI 3.46–10.83; *p* < 0.001). Most of the toilets were dry toilets in both IWMV households (69/83; 83%) and NWMV households (23/27; 85%).

##### Disinfection of Drinking Water Source

There were significant differences in the views of villagers in IWMV and NWMV with respect to the need of disinfection of drinking water at the community water source. Disinfection of the water source throughout the year was viewed more important in IWMV compared to NWMV (85% *vs.* 66%) (OR = 2.81, 95% CI 1.60–4.92; *p* < 0.001).

##### Faecal Coliform Contamination of Drinking Water

Drinking water could be tested for faecal contamination in all the households except four in NWMV. The contamination of water was significantly higher in NWMV compared to IWMV (88% *vs.* 42%, OR = 10.20, 95% CI 5.83–17.85; *p* < 0.001).

##### Diarrhoea

The number of households that reported diarrhoea was significantly higher in NWMV (42/144; 29%) compared to IWMV (17/142; 12%) (OR = 3.02, 95% CI 1.65–5.54; *p* < 0.001).

#### 3.2.3. Cropping Patterns and Choice of Food

The percentage of households that cultivated only rain-fed crops like rice, nagali (*Eleusine* spp.), and bhagar (*Echinochola* spp.) were significantly higher in the NWMV compared to the IWMV (88/136; 65% *vs.* 54/131; 41%). The households that cultivated vegetable crops e.g., eggplant (*Solanum melongena*), green chilli (*Capsicum annum*) and also grew fruit trees, e.g., mango (*Mangifera indica*), cashew-nut (*Anacardium occidentale*) along with rain-fed crops, were significantly higher in IWMV compared to NWMV (77/131; 59% *vs.* 48/136; 35%). The differences in the cropping pattern *i.e.*, rain-fed crops only or rain-fed crops along with fruits and vegetable crops were statistically different between IWMV and NWMV (OR = 2.61, 95% CI 1.60–4.27; *p* < 0.001).

There were no significant differences in the sale of food-grain crops (rice, *nagali* and *bhagar*) between IWMV and NWMV (OR = 1.36, 95% CI 0.82–2.25; *p* = 0.200). Rice and *nagali* were reported as staple foods in all study villages. The proportion of use of fine cereals (rice) as a staple food was significantly higher in IWMV (108/142; 76%) than NWMV (80/144; 56%) (OR = 2.02, 95% CI 1.21–3.36; *p* = 0.007). The households in IWMV reported significantly higher consumption of vegetables (OR = 1.71) and fruits (OR = 1.94) after purchase compared to NWMV. There were no significant differences in the consumption of wild vegetables, eggs, chicken and fish between IWMV and NWMV.

#### 3.2.4. Migration 

Migration occurred from January to May in all six villages. About two-third of the total households reported at least one person migrating in the non-rainy seasons for seasonal employment. About one-third of the total households had to migrate with their families. The number of households with seasonal migration was significantly lower in IWMV (86/142; 61%) compared to NWMV (104/144; 72%) (OR = 0.59, 95% CI 0.36–0.97; *p* = 0.036).

#### 3.2.5. Education of Girls 

A total of 90 households in IWMV and 87 households in NWMV reported having a girl child between 5 and 16 years. For a higher number of girls in IWMV, education did not seem to have been discontinued (80/90; 89%) compared to NWMV (63/87; 72%) (OR = 3.04, 95% CI 1.28–7.64; *p* = 0.005). There was no significant difference in the proportion of boys that were going to the school and those that had left school or never been to school between NWMV and IWMV.

#### 3.2.6. Utilization of Healthcare Services

Table 3 shows that a significantly higher number of households in IWMV utilized government institutional services for antenatal, natal and postnatal care (OR = 3.75, 2.57 and 4.88 respectively) compared to NMWV. Another significant finding was that, in the past three years, there were significantly lower numbers of birth in IWMV (34/142; 24%) compared to NWMV (52/142; 36%) (OR = 0.55, 95% CI 0.33-0.92; *p* = 0.025). A significantly higher number of households from NWMV (19/144; 13%) preferred to go to a traditional healer (*bhagat*) to seek healthcare services for common illnesses compared to IWMV (9/142; 6%) (OR = 2.44, 95% CI 1.08–5.50; *p* = 0.031).

**Table 3:** Differences in the utilization of modern healthcare services at primary health center as reported by the interviewees in six tribal villages, three with and three without implementation of integrated watershed management programme in Thane district, Maharashtra, India.

Variable		IWMV (N = 142) Number (%)	NWMV (N = 144) Number (%)	Odds Ratio	95% Confidence Interval	*p*-Value*
Birth in family in last 3 years	Yes	34 (24)	52 (36)	0.55	0.33–0.92	0.025
	No	108 (76)	92 (64)	Ref		
**Health services utilization**		**(N = 34) ** **Number (%)**	**(N = 52) ** **Number (%)**			
Antenatal care	Yes	18 (53)	12 (23)	3.75	1.49–9.38	0.004
	No	16 (47)	40 (77)	Ref		
Institutional delivery	Yes	25 (74)	27 (52)	2.57	1.01–6.51	0.045
	No	9 (26)	25 (48)	Ref		
Postnatal care	Yes	16 (47)	08 (15)	4.88	1.84–12.98	0.001
	No	18 (53)	44 (85)	Ref		

N—Total number of interviewees; IWMV—Integrated watershed management villages; NWMV—Non-watershed management villages; Ref—reference category; * Chi-square test.

### 3.3. Perceived Impact of IWMP on Public Health from 10-Item Part of the Questionnaire

A higher number of respondents from IWMV (128/142, 90%) had a general understanding of IWMP compared to those in the NWMV (20/144; 14%) (*p* < 0.001). Table 4 indicates that the respondents from the IWMV had significantly higher percentage of positive views on the impact of the IWMP on increased water availability, increased agricultural income, increased employment generation, reduced seasonal migration, increased firewood availability, reduced hard work for women, preserving environment and positive impact on their health and well-being. However, in both the settings, more than 50% respondents perceived that due to IWMP, there was increase in water availability for agriculture use.

**Table 4:** Differences in the views of interviewees on positive impact **^#^** of integrated watershed management programme (IWMP) as measured by 10-item part of the questionnaire on various public health aspects in the six tribal villages, three with and three without implementation of IWMP in Thane district, Maharashtra, India.

Variable	IWMV (N = 142) Number (%)	NWMV (N = 144) Number (%)	*p*-Value *
Increase in water availability	123 (87)	58 (40)	<0.001
Increase in water use for agriculture	61 (43)	53 (37)	0.288
Increase in employment generation	67 (47)	49 (34)	0.023
Increase in income in agriculture	81 (57)	53 (37)	<0.001
Increase in firewood availability	86 (61)	54 (37)	<0.001
Reduction in migration	91 (64)	55 (38)	<0.001
Reduction in hard work of women	110 (78)	57 (40)	<0.001
Reduction in diseases	68 (48)	40 (28)	<0.001
Change in environment	70 (49)	35 (24)	<0.001
Impact on health and well being	131 (92)	79 (55)	<0.001

N—Total number of interviewees; IWMV—Integrated watershed management villages; NWMV—Non-watershed management villages; * Chi-square test; **^#^** response as “yes” to this 10-item part of the questionnaire.

## 4. Discussion

To our knowledge this is the first cross-sectional study on the public health implications of integrated watershed management programmes in general and with respect to a tribal area in particular. The findings of this study indicate that the tribal people in the integrated watershed management villages perceived that the integrated watershed management programme has a positive impact on water availability, hygiene and sanitation practices, cropping pattern, income from agriculture, choice of staple food, migration, utilization of healthcare services and education of girls.

Respondents perceived that due to IWMP, there was increase in water availability for households and agriculture use as well. Our earlier study showed that implementation of IWMP resulted in an increased availability of groundwater, even in water scarce summer season in IWMV [25]. The IWMP recharges ground water by building water harvesting structures, thus enhancing water sustainability [11]. A rise in the water table has been demonstrated in a village in the semi-arid Deccan plateau (which covers most of the southern part of India), where IWMP was implemented [8].

The main objective of the integrated watershed management is to improve water availability for the villagers. Availability of water has great impact on hygiene and sanitation practices of the affected population. Apart from this, the distance of water source and time required for water collection also affect hygiene and sanitation practices that ultimately have impact on health. Literature suggests that, there is a significant increase in illness risk in people living farther away from their water source [26]. The impact of improved water supply and sanitation facilities shows decrease in infections, for e.g., diarrhoea [27]. A major benefit of water supply is the saving of women’s time and effort from water collection. Increase in water use for food preparation after water supply in villages has been reported, which suggests that lack of access to water may also influence diet [28]. The distance to the water source is important in remote areas where women have to spend most of their time in the collection of water. It also becomes hard work for women and affects their health. Thus washing of clothes or daily bathing at river is an indication that women have to spend their productive time in these activities. Literature also suggests that non-availability of water close to houses also affect the bathing of children which may affect their health [28]. Thus washing of clothes at the river may not directly influence health, but is an indicator of lack of access to household water. In tribal villages, mainly women are burdened by everyday water provision responsibility [29]. This hard work can have adverse effects on their health [29]. Results of the present study show that the women had to walk significantly less in IWMV for fetching water compared to NWMV. A study from Mussorie, India showed that the workload of the women can be reduced by up to two hours daily as an impact of watershed management [30]. Girls are also involved in the work of collection of water with their mothers and it can affect their schooling. In IWMV, a lower numbers of households reported discontinuation of school education of girls in comparison to NWMV. Longer duration of water scarcity might have an impact on education of girls.

Open-air defaecation is one important reason for faecal contamination of water in tribal areas [31]. The Government of India has initiated a campaign to create ‘open defaecation free’ villages [23,32]. Perhaps due to this, even in these remote villages, toilets are being constructed and used. In India, water scarcity is one of the reasons for not constructing and using toilets, as cleanup after defecation is water dependent in India. Thus, even in NWMV, there were toilets in some households. However, IWMP facilitated increased water availability in IWMV, thus helping building of toilets and resulting into improved sanitation. Recently the prime minister of India has launched a massive campaign, called “Clean India”, which will ensure that every household has a toilet by the year 2019 [33]. In this light, in water scarce rural and tribal areas of India, more focus on activities like IWMP may be useful to make such campaigns successful.

Water borne diseases are of common occurrence in many tribal areas and this can be prevented by appropriate disinfection of water [34]. The need for disinfection of drinking water wells throughout the year was perceived as important by almost three times greater number of respondents in IWMV compared to NWMV. This supports the findings of our qualitative study in which participants perceived that if they had an assured source of drinking water available for use throughout the year, then they would disinfect the water source regularly [16]. Also, we have previously reported lower contamination of water sources in IWMV compared to NWMV in the same six villages included in the present study [25]. Faecal coliform contamination of even safe water takes place during collection, storage and access in the home [35] and it is since long documented that it becomes one of the reasons for diarrhoeal diseases [36]. Implementation of IWMP and subsequent increased awareness regarding water handling during storage might have resulted in low faecal coliform contamination of household drinking water and lower number of households with occurrence of diarrhoea in IWMV.

Agriculture was the main occupation reported in both settings with mainly food-grain crops, grown for self-consumption. Similar practice has been reported from Bharmaur tribal area of Himachal Pradesh in India [37]. The nutrition of tribal people depends on type of crops cultivated and also on forest produce such as wild vegetables and fruits and malnutrition is a major public health problem in many tribal areas [34]. In the present study, implementation of IWMP appears to have an influence on the choice of food grain, consumption of vegetable and fruits, which is linked to nutrition and health of the population. Therefore, to improve the nutrition in tribal areas more efforts are needed to improve agriculture by changing cropping pattern and increasing crop production. This can be possible through IWMP as it is one of the important objectives of it. Increased net return from agriculture has been demonstrated from other watershed management sites from Thailand, India and China [38]. Adoption of improved agricultural practices might have increased income and thereby increased the capacity of the tribal people to purchase fruits and vegetables for their own consumption as seen in IWMV. In IWMV, about 70% of the households were growing some kind of fruits and vegetables crops; this activity was reported to be less than 50% in NWMV. This indicates that an increase in the availability of water and developmental efforts in IWMP increases the variety of crops cultivated.

Rain-fed agriculture with no other avenues for employment leads to seasonal migration in non-rainy season periods in tribal areas of India and other parts of the world such as Nicargua and Ghana [39,40,41]. When ecosystem services within a watershed do not provide basic material for life or freedom of choice of work, then people have to migrate unwillingly to other places [42]. In resource poor settings, migration is reported as one of the social determinants of health [15]. Multiple physical, psychological and social health problems result from such migration [43,44,45]. In IWMV, migration was significantly lower than in NWMV, probably because of the generation of employment within the village.

In the current study we found that there was greater utilization of government healthcare resources in IWMV. There was also significantly higher percentage of institutional deliveries in IWMV which is an indication of awareness among villagers. The tribal populations are plagued with higher infant and maternal mortality which results from multiple factors like lack of access to healthcare especially emergency care, illiteracy, social customs and beliefs and non-availability of qualified health care providers leading to reliance on traditional healers [46,47,48]. People’s participation is the key feature of IWMP which leads to the effectiveness of the programme. Creation of self-help groups during the implementation of IWMP helps to strengthen local institutions and also helps to increase awareness regarding developmental aspects including health. Inclusion of such activities in IWMP may lead to socio-economic empowerment of people further leading to an increased awareness and demand for modern healthcare. Also lower number of births in IWMV might be due to higher awareness regarding family planning.

The strength of our study is that it had a high response rate (92%). Another strength of the study is the use of a comprehensive questionnaire for the collection of data that covered most potential public health aspects linked to the IWMP. The study was conducted in a hilly tribal area, as development there is dependent on water and a small change in the situation can have a noticeable impact. The limitation of our study is that it presents the practices of households as reported by the household head. Actual practices might differ among household members. There are also chances that factors such as recall bias [49,50] or temporary situations might have had an influence on the responses by the respondents. These limitations were taken into considerations while interpreting the findings and it was also assumed that effect of recall bias or temporary situations would not vary considerably between the two settings. Besides that, the findings of this paper are based on the information reported by the household heads and the data was not verified by any other institutional records. 

We also assessed the faecal contamination of drinking water at each household using a hydrogen sulfide (H_2_S) test, which is a rapid, simple, inexpensive test for testing the microbial quality of drinking water. This test is widely used and is recommended for small community and household water supplies in remote areas [51] and therefore was considered suitable for the present study. The test detects the production of H_2_S in a volume of water by enteric bacteria associated with fecal contamination by the formation of a black precipitate from the reaction of the H_2_S with iron in the medium. However, the chances of production of H_2_S by other factors such as non-fecal bacteria or abiotic factors cannot be totally excluded.

## 5. Conclusions

The results of the cross-sectional study indicate that implementation of an integrated watershed management programme in tribal areas positively impacts the water availability especially in non-rainy seasons, and improves hygiene and sanitation by making water resources available for bathing, washing and for toilet use. It also indicates that such an integrated watershed management programme facilitates improvement in cropping pattern and makes available more fine cereals, vegetables and fruits for consumption and help to decrease migration. Implementation of integrated watershed management programmes may contribute to improvement of public health in a hilly tribal area. To elucidate this further, more definitive larger studies are needed.
